# Evaluation of radically open dialectical behaviour therapy in an adult community mental health team: effectiveness in people with autism spectrum disorders

**DOI:** 10.1192/bjb.2020.113

**Published:** 2021-06

**Authors:** Peter L. Cornwall, Susan Simpson, Claire Gibbs, Valerie Morfee

**Affiliations:** 1 Redcar & Cleveland Mental Health Services, Tees, Esk and Wear Valleys NHS Foundation Trust, UK

**Keywords:** Radically open dialectical behaviour therapy, autism spectrum disorders, community mental health teams, group psychotherapy, outcome studies

## Abstract

**Aims and method:**

Radically open dialectical behaviour therapy (RO DBT) is a transdiagnostic treatment designed to address disorders associated with overcontrol, including autism spectrum disorders (ASD). To date, no studies have reported on the effectiveness of RO DBT for people with ASD. Forty-eight patients were referred to a RO DBT programme, of whom 23 had a diagnosis of ASD. Outcome was measured using the Clinical Outcomes in Routine Evaluation – Outcome Measure (CORE) and the Questionnaire about the Process of Recovery.

**Results:**

The intervention was effective, with a medium effect size of 0.53 for improvement in CORE global distress. End-point CORE global distress score was predicted from initial severity and a diagnosis of ASD. Participants with a diagnosis of ASD who completed the therapy had significantly better outcomes than completing participants without an ASD diagnosis.

**Clinical implications:**

These findings provide preliminary support for RO DBT as an effective intervention for ASD in routine settings.

Society generally holds self-control in high esteem and it can lead to better health outcomes.^1^ Consequently, high self-control has not commonly been a focus for psychological intervention. However, too much self-control, or maladaptive overcontrol, is associated with a range of problems, including social isolation, poor interpersonal functioning and mental illnesses such as chronic depression, anorexia nervosa and obsessive–compulsive disorder.^2^ Radically open dialectical behaviour therapy (RO DBT) has been developed as an adapted form of dialectical behaviour therapy to directly target overcontrol, and is now supported by several controlled clinical trials.^3^ The therapy introduces strategies to improve social connectedness and intimacy and to reduce social isolation by improving emotional expression and the ability to respond more flexibly. The primary mechanism of change is through social signalling.^4^ In RO DBT, what matters most is how a person communicates or socially signals their inner experiences to others and the impact that social signalling has on their experience of social connectedness. RO DBT has been delivered in a variety of clinical settings, but there are no published studies of the effectiveness of RO DBT delivered in an adult community mental health team (CMHT).

Autism spectrum disorders (ASD) have also been recognised as disorders of maladaptive overcontrol, but ASD have not been recorded as comorbid conditions in the studies of RO DBT to date.^4^ In the UK, the Autism Act 2009 requires National Health Service (NHS) trusts to provide access to services for the diagnosis of autism in adults and this has resulted in a large increase in referrals of individuals seeking this diagnosis to explain their difficulties.^5^ However, the National Institute for Health and Care Excellence (NICE) guideline on the management of ASD in adults indicates very limited evidence for psychosocial interventions.^6^

This study reports on the effectiveness of RO DBT for people with maladaptive overcontrol in an NHS adult CMHT setting, with a specific focus on the outcomes of a subsample of these individuals who are diagnosed with ASD.

## Method

### Setting

The study was undertaken in an NHS secondary care CMHT in northern England providing care and treatment for patients with mainly non-psychotic disorders. The RO DBT programme was open to other adult patients in the trust because it was the only service offering RO DBT, but the vast majority of patients came from the local team. The study used data collected routinely for all patients receiving group therapy in the service and so did not constitute research requiring ethical approval or informed consent from participants. The study was approved by Tees, Esk and Wear Valleys NHS Foundation Trust.

### Sample

Patients were considered eligible if their lead clinician considered that they were presenting with maladaptive overcontrol in the context of their mental health problems or following a team case formulation meeting. Patients were identified as meeting criteria for overcontrol using a clinical interview and a range of tools, including the Assessing Styles of Coping: Word-Pair Checklist, the OC Trait Rating Scale, and the Brief Overcontrol Scale.^2,7,8^ We only accepted patients who acknowledged that they had a coping style characterised by overcontrol and that they wanted to change it. Patient diagnosis was identified from the patient electronic record.

### Intervention

The typical RO DBT out-patient format is a 30-week programme and involves a 1 h weekly individual session and a 2.5 h weekly skills training session. The primary goal is to decrease behavioural overcontrol and aloofness, rather than decrease behavioural dyscontrol and mood-dependent responding, as in standard DBT.^9^ Patients are encouraged to practice disinhibition, participate without planning and to be more emotionally expressive.^2,4^ In this study, regular individual sessions were not possible because of the limited staffing resource in the CMHT. Occasionally, participants received additional individual sessions, for example, if they started late in the programme as a means of catching up. Delivering a group-only programme is a recognised variation to the standard approach.^10–12^

Five cohorts of patients over the course of 3 years participated in a RO DBT programme consisting of 30 weekly skills training classes. Some started the programme halfway through a cohort and so continued into the next cohort. The fifth cohort programme was cut short by the COVID-19 pandemic, when all group therapy programmes were suspended.

### Therapists

Four psychological therapists trained in RO DBT delivered the programme (three psychiatric nurses and one occupational therapist) and they were supported by one psychiatric nurse in co-facilitating some of the skills classes. Clinical supervision was provided during weekly consultation meetings using the model of standard DBT.^9^

### Measures

In the first skills class, participants completed the Clinical Outcomes in Routine Evaluation – Outcome Measure (CORE)^13^ and the Questionnaire about the Process of Recovery (QPR).^14^ These measures were repeated at mid-point (week 15) and end-point (week 30).

The CORE is a 34-item self-report questionnaire measuring the level of global distress the person has experienced in the previous week, measured on a five-point scale ranging from 0 to 4, and reported as a mean score per item. The measure contains four subdomains: subjective well-being, problems or symptoms, social and life functioning, and risk of harm to self and others. It has become a standard tool for measuring outcome in psychological therapy studies and has good psychometric properties.^13,15^ The recommended clinical cut-off for CORE global distress is a mean item score of 1.0, with scores above this threshold considered to represent ‘clinical caseness’. A mean item score of 2.0 represents moderately severe distress. Reliable change is indicated by a global distress score change of at least 0.5, whereas clinical recovery is indicated by a reduction in the mean global distress score to <1.0.^16^

The QPR is a 25-item self-report measure designed to evaluate the achievement of recovery goals in severe mental illness. It was designed collaboratively by clinicians and patients and has been recommended as a tool to promote engagement and a collaborative clinical approach, but also as a method of detecting change in recovery in CMHTs.^14,17,18^ As such, it can measure whether the service is meeting the perceived needs of patients irrespective of their clinical outcome.

### Data analysis

Data analysis was undertaken using Real Statistics for Excel 365 for Windows.^19^ Baseline characteristics were described comparing differences between those completing the programme to 30 weeks (the per-protocol sample) and those dropping out early or not attending at all (the ITT sample). For the intention-to-treat (ITT) analyses we used the last observation carried forward (LOCF) method. Improvement in outcome measures at 15 and 30 weeks was examined for per-protocol (*n* = 21) and ITT (*n* = 35) samples using analysis of variance (ANOVA). Effect sizes were calculated using Cohen's *d* with 95% confidence intervals.^20^ Predictors of outcome at 30 weeks were examined for per-protocol and ITT samples using stepwise multiple linear regression. As a *post hoc* analysis, we compared the ASD and non-ASD participants for per-protocol and ITT samples for continuous outcomes using the *t*-test and categorical outcomes using the χ^2^-test.

## Results

### Sample characteristics

Of the 48 eligible participants, 23 had a confirmed or working diagnosis of ASD, either as the only identified problem (*n* = 3) or as a comorbid condition (*n* = 20). This was the single most common diagnosis among the participants. Thirteen patients from this group had the diagnosis confirmed by the local specialist autism service; the other ten were on the waiting list for specialist assessment, but the working diagnosis was an autism spectrum condition. The primary diagnoses were depressive disorder (*n* = 14), generalised anxiety disorder (*n* = 8), bipolar disorder (*n* = 6), personality disorder (*n* = 5), post-traumatic stress disorder (*n* = 4), schizophrenia and related disorders (*n* = 4), anorexia nervosa (*n* = 3), ASD (*n* = 3) and attention-deficit hyperactivity disorder (*n* = 1).

### Participant flow

**Fig. 1:**
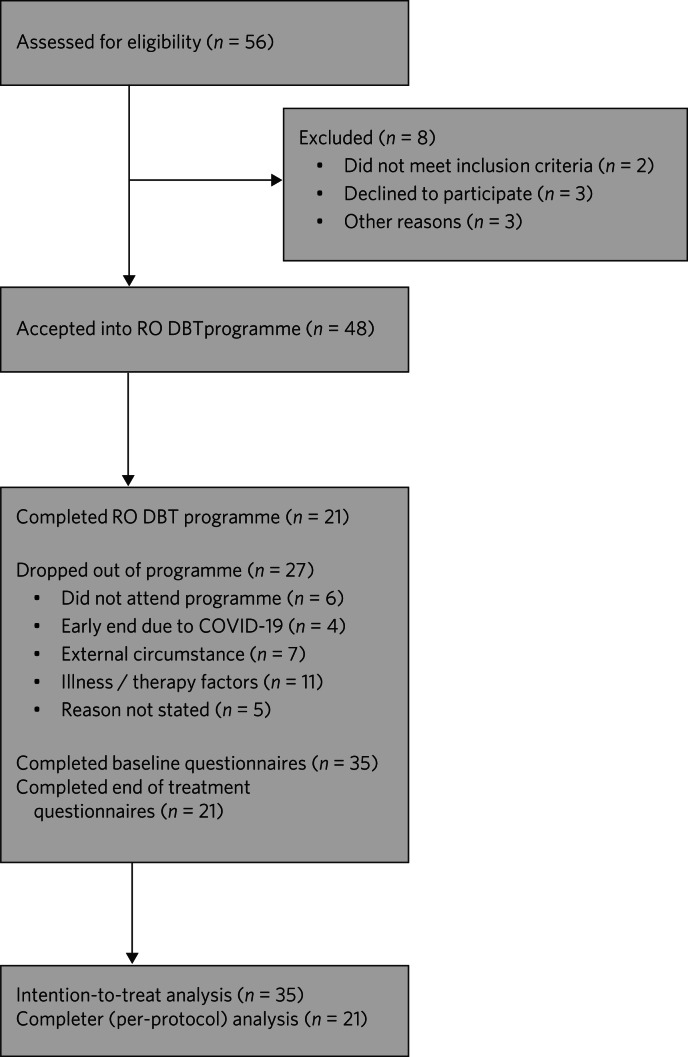
CONSORT flow diagram. RO DBT, radically open dialectical behaviour therapy.

The flow of participants through the study is shown in Fig. 1. In total, 21 participants completed the programme with outcome data recorded at mid-point (week 15) and at end-point (week 30). Participants in the fifth cohort were able to complete only 15 sessions before the sessions were stopped because of the COVID-19 pandemic and so their data are included in the ITT analysis but not the per-protocol analysis.

Non-attenders and those who dropped out did not differ from completers with respect to age, gender, ASD diagnosis or global severity of problems but reported a higher level of risk and a lower QPR score at baseline (Table 1). The sample reflected the CMHT's case-load, with more women than men and overwhelmingly White British in ethnic origin.

**Table 1:** Participant characteristics according to treatment completion

	Treatment completers (*n* = 21)		Treatment non-completers (*n* = 27)		
	*n*	%	*n*	%	Comparison between groups^a^
Female	13	61.90	14	51.85	χ^2^ = 0.31, d.f. = 1*, P* = 0.58
White British	21	100.00	27	100.00	*P* = 1.00
Autism diagnosis	11	52.38	12	44.44	χ^2^ = 0.13, d.f. = 1*, P* = 0.72
	Mean	s.d. (range)	Mean	s.d. (range)	
Age, years	40.67	13.33 (20–58)	36.11	13.05 (18–59)	*t* = 0.71, d.f. = 47, *P* = 0.24
Sessions attended	25.81	3.22 (20–30)	5.67	5.26 (0–18)	*t* = 14.98, d.f. = 47, *P* **<** **0.001**
Baseline CORE global distress score	2.06	0.66 (0.68–3.06)	2.48	0.81 (0.76–3.24)	*t* = 1.13, d.f = 34, *P* = 0.10
Baseline CORE well-being score	2.44	0.75 (0.50–3.50)	3.02	0.98 (1.00–4.00)	*t* = 1.62, d.f. = 34, *P* = 0.06
Baseline CORE problems score	2.52	0.86 (0.75–3.67)	2.93	0.85 (1.00–3.75)	*t* = 0.97, d.f. = 34, *P* = 0.17
Baseline CORE functioning score	2.19	0.67 (1.00–3.17)	2.46	0.84 (0.75–3.42)	*t* = 0.53, d.f. = 34, *P* = 0.30
Baseline CORE risk score	0.64	0.65 (0.00–2.50)	1.27	0.87 (0–2.33)	*t* = 2.16, d.f. = 34, ***P*** **=** **0.02**
Baseline QPR score	24.67	9.19 (4–44)	15.27	11.86 (0–34)	*t* = 2.06, d.f. = 31, ***P*** **=** **0.02**

CORE, Clinical Outcomes in Routine Evaluation – Outcome Measure; QPR, Questionnaire about the Process of Recovery.

a. χ^2^ for frequency variables, *t*-test for continuous variables.

Bold denotes significance at *P* < 0.05.

The mean CORE global distress score at baseline was >2.0 for both groups, indicating that participants had moderately severe mental health problems. The lower score on the QPR measure in treatment non-completers suggests that this group was less well engaged with their care and treatment at the outset.

The reasons for drop out are reported in Fig. 1. External circumstances included starting a new job and taking on childcare responsibilities. Illness/therapy factors included feeling too unwell to continue participation and not being able to grasp the concepts discussed in the sessions.

Participants with a diagnosis of ASD did not differ from those without an ASD diagnosis with respect to mean age (35.6 *v.* 40.2 years, *t* = 0.69, d.f. = 47, *P* = 0.30), female gender (52 *v.* 60%, χ^2^ = 0.30, d.f.= 1*, P* = 0.59), mean baseline CORE global distress score (2.39 *v.* 2.06, *t* = 0.97, d.f. = 34, *P* = 0.18) or mean baseline QPR score (21.73 *v.* 21.18, *t* = 1.27, d.f. = 31, *P* = 0.89).

### Outcomes at 30-week end-point for per-protocol and ITT samples

The intervention was effective in both the per-protocol (*n* = 21) and the ITT (*n* = 35) samples (Table 2). In the per-protocol sample, five participants (24%) achieved a CORE global distress score indicative of clinical recovery (score <1.0) and nine (43%) made a reliable improvement (reduction in score >0.5). The effect size for change in CORE global distress was medium to large (Cohen's *d* = 0.59). The mean improvement at the 30-week end-point was 0.43 (95% CI 0.09–0.78) for the CORE global distress and the mean increase in QPR score was 8.29 (95% CI 3.00–13.57).

**Table 2:** Per-protocol and intention-to-treat (ITT) analyses of outcomes

	Baseline, mean (s.d.)	Mid-point, mean (s.d.)	Final, mean (s.d.)	*F*	d.f.	*P*	Effect size (95% CI for *d*)
Per-protocol sample (*n* = 21)							
CORE global distress score	2.06 (0.66)	1.78 (0.78)	1.63 (0.81)	6.02	20	**<0.01**	0.59 (0.57–0.61)
CORE well-being score	2.44 (0.75)	2.12 (1.05)	1.92 (1.00)	4.74	20	**0.01**	0.59 (0.57–0.61)
CORE problems score	2.52 (0.86)	2.15 (0.96)	2.00 (0.97)	6.55	20	**<0.01**	0.57 (0.55–0.59)
CORE functioning score	2.19 (0.67)	1.85 (0.71)	1.74 (0.83)	5.62	20	**0.01**	0.59 (0.57–0.61)
CORE risk score	0.64 (0.65)	0.68 (0.77)	0.46 (0.68)	2.48	20	0.10	0.27 (0.25–0.29)
QPR score	24.67 (9.91)	29.68 (11.83)	32.95 (11.59)	11.61	20	**<0.001**	0.77 (0.75–0.79)
ITT sample (*n* = 35)							
CORE global distress score	2.23 (0.74)	1.98 (0.83)	1.85 (0.93)	8.26	34	**<0.001**	0.46 (0.45–0.48)
CORE well-being score	2.67 (0.88)	2.46 (1.11)	2.23 (1.18)	4.35	34	**0.02**	0.40 (0.39–0.42)
CORE problems score	2.69 (0.87)	2.36 (1.01)	2.21 (1.12)	9.32	34	**<0.001**	0.47 (0.45–0.48)
CORE functioning	2.30 (0.74)	2.00 (0.76)	1.93 (0.87)	8.65	34	**<0.001**	0.47 (0.46–0.49)
CORE risk score	0.90 (0.80)	0.86 (0.80)	0.68 (0.79)	3.31	34	**0.04**	0.27 (0.26–0.29)
QPR score	21.44 (11.37)	25.59 (13.67)	28.23 (14.86)	14.86	31	**<0.001**	0.52 (0.50–0.53)

CORE, Clinical Outcomes in Routine Evaluation – Outcome Measure; QPR, Questionnaire about the Process of Recovery.

Bold denotes significance at *P* < 0.05.

In the ITT sample, 9 participants (26%) achieved a CORE global distress score indicative of clinical recovery and 13 (37%) made a reliable improvement. The effect size for change in CORE global distress was small to medium (Cohen's *d* = 0.46). The mean improvement at the 30-week end-point was 0.38 (95% CI 0.02–0.74) and the mean increase in QPR was 6.69 (95% CI 0.33–13.05).

Seven participants (five with an ASD diagnosis) continued in the therapy group beyond 30 weeks for clinical reasons. The mean improvement for all participants (*n* = 28) with outcome at the end of their intervention was 0.53 (95% CI 0.24–0.82) on the CORE global distress score, which represents a clinically reliable improvement overall.

### Predictors of outcome

We used stepwise regression to examine which factors predicted the outcome score at 30 weeks in participants completing the programme to 30 weeks (per-protocol, *n* = 21) and the intention-to-treat sample (ITT, *n* = 35). We entered the following variables into the analysis: age, gender, initial severity (CORE global distress score at baseline), diagnosis of ASD, cohort and number of sessions attended.

Initial severity and diagnosis of ASD were entered into the model in both per-protocol and ITT samples and accounted for a highly significant amount of the variation in the final outcome score – 60% in the per-protocol sample and 55% in the ITT sample (Table 3). Participants with lower baseline CORE global distress scores and a diagnosis of autism were significantly more likely to have a better final outcome score.

**Table 3:** Predictors of final Clinical Outcomes in Routine Evaluation – Outcome Measure (CORE) global distress score

Model					*P*
Per-protocol sample (*n* = 21)					
	*r*^2^ = 0.64	adj. *r*^2^ = 0.60		*F* = 15.76	**<0.001**
Variables	β	s.e.	95% CI	*t*	
Constant	0.27	0.39			
Initial severity	0.85	0.18	0.54–1.15	4.81	**<0.001**
Autism	0.75	0.23	0.34–1.13	3.27	**<0.01**
Intention-to-treat sample (*n* = 35)					
	*r*^2^ = 0.57	adj. *r*^2^ = 0.55		*F* = 21.65	**<0.001**
Variables	β	s.e.	95% CI	*t*	
Constant	0.08	0.33			
Initial severity	0.95	0.14	0.70–1.19	6.58	**<0.001**
Autism	0.37	0.21	0.01–0.73	1.76	0.09

Bold denotes significance at *P* < 0.05.

### Comparison between ASD and non-ASD participants at the end of treatment for continuous and categorical outcomes

Participants with a confirmed or working diagnosis of ASD showed clinically reliable improvement and showed better outcomes than non-ASD participants with respect to functioning and perception of recovery (Table 4).

**Table 4:** Comparison of change in continuous outcomes between participants with and without a diagnosis of autism spectrum disorder (ASD)

	Change in ASD (s.d.)	Change in non-ASD (s.d.)	*T*	d.f.	*P*
Per-protocol sample^a^					
CORE global distress score	0.79 (0.61)	0.04 (0.37)	3.04	20	**<0.01**
CORE well-being score	0.89 (0.90)	0.13 (0.58)	1.93	20	**0.03**
CORE problems score	0.94 (0.60)	0.06 (0.52)	3.28	20	**<0.01**
CORE functioning score	0.83 (0.79)	0.03 (0.43)	2.47	20	**0.01**
CORE risk score	0.38 (0.45)	−0.03 (0.36)	1.96	20	**0.03**
QPR score	12.18 (6.97)	4.00 (7.29)	2.29	20	**0.02**
ITT sample^b^					
CORE global distress score	0.57 (0.57)	0.18 (0.62)	1.57	34	0.06
CORE well-being score	0.54 (0.84)	0.28 (0.97)	0.26	34	0.40
CORE problems score	0.67 (0.61)	0.24 (0.77)	1.46	34	0.08
CORE functioning score	0.62 (0.69)	0.12 (0.55)	2.00	34	**0.03**
CORE risk score	0.31 (0.38)	0.12 (0.59)	0.62	34	0.27
QPR score	9.53 (7.73)	4.18 (8.74)	1.46	31	0.08

CORE, Clinical Outcomes in Routine Evaluation – Outcome Measure; QPR, Questionnaire about the Process of Recovery.

a. Per-protocol sample: ASD, *n* = 11; non-ASD, *n* = 10.

b. Intention-to-treat (ITT) sample: ASD, *n* = 18; non-ASD, *n* = 17.

Bold denotes significance at *P* < 0.05.

At the end of treatment the ASD and non-ASD participants (*n* = 28) did not differ significantly in the number who met the clinical recovery threshold (CORE global distress score <1.0) (χ^2^ = 2.01, d.f. = 1*, P* = 0.16). However, participants with ASD were significantly more likely to have a reliable improvement in CORE global distress score in the per-protocol but not the ITT sample. In the per-protocol sample, 73% of participants with ASD showed reliable improvement, compared with 10% of non-ASD participants (χ^2^ = 8.21, d.f. = 3*, P* = 0.04). In the ITT sample, the figures were 56% for those with ASD and 18% for non-ASD participants (χ^2^= 4.58, d.f. = 3*, P* = 0.21).

Among the male participants completing the programme, 4/8 (50%) achieved clinical recovery (a CORE global distress score <1.0) compared with 2/13 (15.4%) of the female participants. However, the difference between men and women was not statistically significant (χ^2^ = 2.41, d.f. = 1*, P* = 0.12).

## Discussion

The findings from this study provide preliminary evidence for the effectiveness of RO DBT for mental disorders in routine clinical practice, and in particular for adults with ASD without intellectual disability. RO DBT is a treatment for maladaptive overcontrol, which characterises many individuals with autism, and demonstrating that RO DBT is a potentially effective treatment for this population is therefore an important step forward.

There have been three randomised controlled trials of RO DBT for refractory depression^21–23^ and two open trials for anorexia nervosa.^24,25^ The only published study in a mixed diagnostic group is a non-randomised controlled trial in adults with mental health problems related to overcontrol referred to a specialist psychological therapy service.^10^ RO DBT has been delivered in a variety of clinical settings, including psychological therapy services, US military veterans services, eating disorder services and forensic in-patient care.^12^

In psychiatric settings, ASD is almost certainly underdiagnosed as a comorbid difficulty or misdiagnosed as a psychotic disorder, personality disorder or obsessive–compulsive disorder,^26,27^ and adults with ASD have high levels of psychiatric comorbidity and dysfunction.^28,29^ ASD is also a significant risk factor for suicidal behaviour.^30^ Previous studies on treatment for ASD have generally looked at adapting established psychological therapies to treat comorbid conditions in people with ASD as a way of improving their mental health and well-being.^31,32^ There have been very few studies designed to improve functioning in autism itself,^33,34^ and the current NICE recommendations are largely extrapolated from work in adolescent and intellectual disability populations.^6^

Our results can best be compared with two open trials that also used the CORE as a primary outcome measure. A study of modified individual CBT for people with ASD in a specialist psychological therapy service had a larger sample (*n* = 81), but with milder baseline severity (mean CORE global distress 1.79).^35^ Of the participants completing the therapy in that study, 37% showed reliable improvement and 19% achieved clinical recovery on the CORE global distress score, compared with 73 and 36% respectively for the participants with ASD in our study. A study of RO DBT in in-patients with anorexia nervosa had a sample size of 47 and a baseline severity of 2.21 for CORE global distress, very similar to our study.^24^ The mean change in CORE global distress in the ITT sample was 0.46, with an effect size of 0.71, thereby showing a more positive outcome overall compared with the current study.

Social signalling, such as social smiling and prosocial body language, has been regarded as a core deficit in ASD that is not readily amenable to change. Our anecdotal impression was that many participants with ASD were able to learn social signalling, and several gave positive feedback. For example one anonymous participant commented, ‘I cannot believe how much it has helped me understand myself, ASD and how to approach the problems I face with the condition’. Given that the treatment is designed to treat overcontrol irrespective of ASD diagnosis, our finding that participants with ASD gained greater benefit requires further exploration.

### Strengths and limitations

For any treatment of ASD to be delivered, it must be feasible within the current clinical practice of mental health service delivery. One of the strengths of this study is that it was implemented in a government-funded community treatment team, alongside the routine delivery of care and treatment for a wide range of mental health conditions, and without any additional resources. It is also important to note that the participants treated had an illness of at least moderate severity and were not selected for the likelihood of responding to the treatment.

There are several limitations that we recognise. This was an uncontrolled study and we did not gather data on what other treatment participants might have received. The study sample was entirely White British, so the findings may not be translatable to multi-ethnic populations. The sample size is small, and a large proportion of participants did not complete the 30 weeks of the RO DBT programme. This high attrition rate can at least partly be explained by an apparent lack of motivation and engagement at baseline among the treatment non-completers, as demonstrated by their lower QPR scores. In addition, we did not measure the fidelity of the programme to the RO DBT manual. However, despite the fact that participants in this study received a more limited version of the recommended RO DBT standard of one-to-one therapy in addition to weekly skills classes, positive findings were found in both the per-protocol and the intention-to-treat samples. This in itself is important to note, as most government-funded community treatment centres, like the one in this study, would not have the resources to provide the full programme of RO DBT. Our outcome measures were limited to self-report questionnaires, which might have been biased in favour of the treatment received, and we do not know whether the positive outcomes experienced by the participants were sustained beyond the 30-week therapy programme. We did not record the severity of overcontrol at baseline, which is one possible explanation for the better outcome in participants with an ASD diagnosis.

### Implications for future research

Future research needs to examine RO DBT in ASD using a randomised controlled trial methodology and it would be important to record any additional psychological and pharmacological treatments that participants receive for comorbid conditions. Follow-up beyond the end of the therapy programme should also be measured. There are already modified versions of RO DBT being delivered^11^ and so studies need to carefully describe the content of the therapeutic intervention and compare more and less intensive models of RO DBT. Studies using RO DBT need to report the number of participants with ASD where the treatment is directed at other disorders, as ASD is likely to be a comorbid condition in many disorders of overcontrol.

## Data Availability

The data that support the findings of this study are available from the corresponding author, [PLC], upon reasonable request.

## About the authors

**Peter L. Cornwall** is a consultant psychiatrist with Redcar & Cleveland Mental Health Services, Tees, Esk and Wear Valleys NHS Foundation Trust, UK. **Susan Simpson** is a psychological therapist with Redcar & Cleveland Mental Health Services, Tees, Esk and Wear Valleys NHS Foundation Trust. **Claire Gibbs** is a clinical nurse specialist with Redcar & Cleveland Mental Health Services, Tees, Esk and Wear Valleys NHS Foundation Trust. **Valerie Morfee** is a team manager with Redcar & Cleveland Mental Health Services, Tees, Esk and Wear Valleys NHS Foundation Trust.
