# Evaluation of radically open dialectical behaviour therapy in an adult community mental health team: effectiveness in people with autism spectrum disorders – ERRATUM

**DOI:** 10.1192/bjb.2022.47

**Published:** 2022-10

**Authors:** Peter L. Cornwall, Susan Simpson, Claire Gibbs, Valerie Morfee

**Keywords:** Radically open dialectical behaviour therapy, autism spectrum disorders, community mental health teams, group psychotherapy, outcome studies

The above article was published with incorrect terms stating ‘gender’ when the correct term was ‘sex’. This has now been updated in the online PDF and HTML versions of the article.

The Publisher apologises for the error.
